# Interplay of *Trans*- and *Cis*-Interactions of Glycolipids in Membrane Adhesion

**DOI:** 10.3389/fmolb.2021.754654

**Published:** 2021-11-19

**Authors:** Batuhan Kav, Bruno Demé, Christian Gege, Motomu Tanaka, Emanuel Schneck, Thomas R. Weikl

**Affiliations:** ^1^ Max Planck Institute of Colloids and Interfaces, Department of Theory and Bio-Systems, Potsdam, Germany; ^2^ Institut Laue-Langevin, Large Scale Structures Group, Grenoble, France; ^3^ Heidelberg University, Institute of Physical Chemistry of Biosystems, Heidelberg, Germany; ^4^ Kyoto University, Institute for Advanced Study, Center for Integrative Medicine and Physics, Kyoto, Japan; ^5^ Max Planck Institute of Colloids and Interfaces, Department of Biomaterials, Potsdam, Germany; ^6^ Technische Universität Darmstadt, Physics Department, Darmstadt, Germany

**Keywords:** glycolipids, carbohydrate–carbohydrate interactions, LewisX carbohydrate, membrane adhesion, membrane shape fluctuations, molecular dynamics (MD) simulations, neutron scattering

## Abstract

Glycolipids mediate stable membrane adhesion of potential biological relevance. In this article, we investigate the *trans*- and *cis*-interactions of glycolipids in molecular dynamics simulations and relate these interactions to the glycolipid-induced average separations of membranes obtained from neutron scattering experiments. We find that the *cis*-interactions between glycolipids in the same membrane leaflet tend to strengthen the *trans*-interactions between glycolipids in apposing leaflets. The *trans*-interactions of the glycolipids in our simulations require local membrane separations that are significantly smaller than the average membrane separations in the neutron scattering experiments, which indicates an important role of membrane shape fluctuations in glycolipid *trans*-binding. Simulations at the experimentally measured average membrane separations provide a molecular picture of the interplay between glycolipid attraction and steric repulsion of the fluctuating membranes probed in the experiments.

## 1 Introduction

Glycolipids are abundant components of biological membranes and play important roles in cell–cell interactions (Schnaar, 2004; Day et al., 2015; Varki, 2017; Poole et al., 2018) and the interactions of stacked membranes in cellular organelles (Stoffel and Bosio, 1997; Boudiere et al., 2014). Besides glycolipid recognition by proteins (Liu and Rabinovich, 2005; Arnaud et al., 2013), glycolipid–glycolipid interactions have been investigated in a variety of reconstituted or synthetic systems including nanoparticles and surfaces functionalized with carbohydrate tips of glycolipids (de la Fuente et al., 2001; Hernáiz and de la Fuente, 2002; de la Fuente et al., 2005), atomic force microscopy setups (Tromas et al., 2001; Bucior et al., 2004; Lorenz et al., 2012; Witt et al., 2016), reconstituted vesicles (Pincet et al., 2001; Gourier et al., 2005; Kunze et al., 2013), as well as supported membranes (Yu et al., 1998), and stacks of membranes (Schneck et al., 2011; Latza et al., 2020) containing glycolipids. Experiments with giant vesicles and stacks of membranes indicate that glycolipids can mediate stable membrane adhesion (Gourier et al., 2005; Schneck et al., 2011; Latza et al., 2020), but a molecular view and quantification of the glycolipid–glycolipid interactions that lead to membrane adhesion is still largely missing.

**FIGURE 1:**
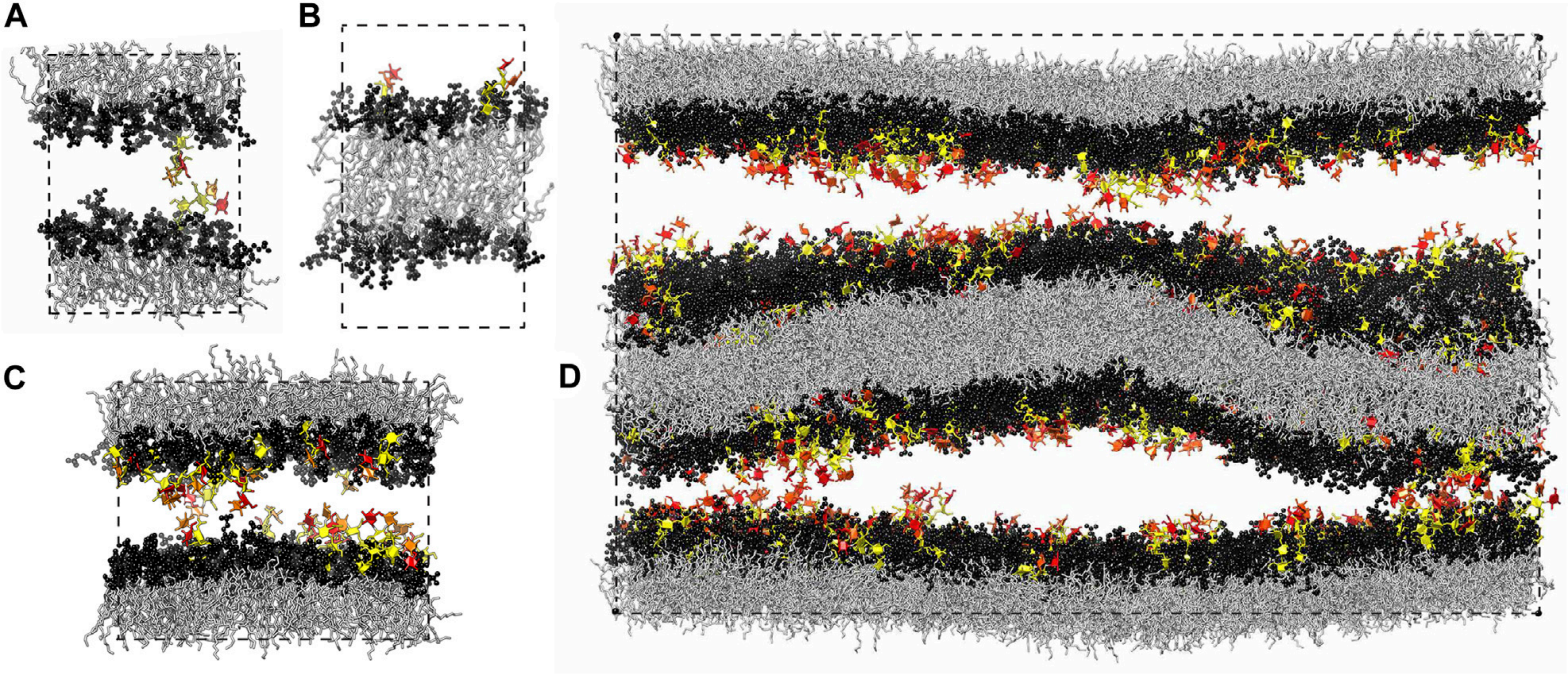
Simulation systems composed of **(A)** a single membrane with 35 lipids and 1 glycolipid in each leaflet, **(B)** a single membrane with 36 lipids in one leaflet and 34 lipids and 2 glycolipids in the other leaflet, **(C)** a single membrane with 90 lipids and 10 glycolipids in each leaflet, and **(D)** two membranes with 810 lipids and 90 glycolipids in each of the four leaflets. The lipids in all our simulation systems are phosphatidylcholine (POPC), and the glycolipids in the simulation snapshots shown here are Le^X^ glycolipids. The fucose and galactose at the branched tip of the Le^X^ glycolipids are represented in red and orange, and the remaining three monosaccharide units are in yellow. The dashed lines illustrate the simulation boxes with periodic boundaries.

In this article, we present detailed results for the *trans*- and *cis*-interactions between glycolipids in membrane adhesion from atomistic molecular dynamics (MD) simulations and relate these interactions to the glycolipid-induced average separations of membranes obtained from neutron scattering experiments. Previously reported results for the *trans*-interaction of a single Lewis^X^ (Le^X^) glycolipid pair obtained in the simulation system of Figure 1A led to membrane adhesion energies mediated by Le^X^ glycolipids and maximally sustained forces of *trans*-complexes of Le^X^ glycolipids in good agreement with experimental results (Kav et al., 2020), which indicates that our simulations provide a realistic picture of glycolipid interactions in membrane adhesion. Here, we extend these previous simulation results by quantifying the *trans*-interactions between glycolipids embedded in apposing membrane leaflets and the *cis*-interactions of glycolipids embedded in the same membrane leaflet in a variety of simulation systems (see Figure 1). We find that *cis*-interactions of glycolipids tend to strengthen the *trans*-interactions and that the *trans*-interactions of the glycolipids in our simulations occur at local membrane separations that are significantly smaller than the average membrane separations in the neutron scattering experiments. Simulations at these average separations in the system of Figure 1D provide a molecular picture of the role of membrane shape fluctuations in glycolipid interactions probed in the experiments.

## 2 Methods

### 2.1 Simulation Systems

**FIGURE 2:**
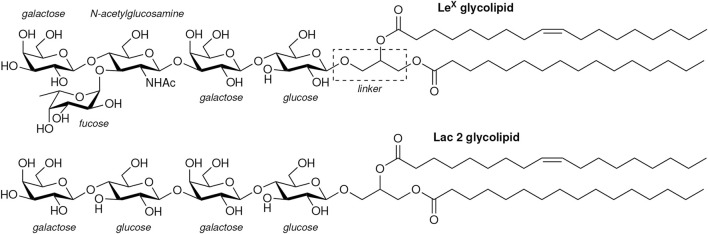
Structures of the Le^X^ and Lac 2 glycolipids investigated in our simulations.

We have investigated the interactions of Le^X^ and of Lac 2 glycolipids in simulations in which these glycolipids are embedded in POPC lipid membranes. Our Le^X^ and Lac 2 glycolipids have the same lipid tails as POPC and carbohydrate tips that are connected to these lipid tails by a glycerol linker group (see Figure 2). Standard carbohydrate force fields overestimate attractive carbohydrate–carbohydrate interactions, which leads to osmotic pressures for solutions of neutral carbohydrates that are systematically too low compared to experimental values (Lay et al., 2016; Sauter and Grafmüller, 2016). We have therefore used the ${GLYCAM06}_{\text{OSMOr}14}^{\text{TIP}5\text{P}}$ force field for the carbohydrate tips of our glycolipids, in combination with the standard AMBER Lipid14 force field (Dickson et al., 2014) for the glycolipid tails and the POPC lipids (Kav, 2019; Kav et al., 2020). In the ${GLYCAM06}_{\text{OSMOr}14}^{\text{TIP}5\text{P}}$ force field, the van der Waals parameters for carbohydrate–carbohydrate interactions of the standard force field GLYCAM06 have been reparametrized to correctly reproduce experimentally measured osmotic pressures (Sauter and Grafmüller, 2016). The ${GLYCAM06}_{\text{OSMOr}14}^{\text{TIP}5\text{P}}$ force field employs the TIP5P water model that leads to more reliable carbohydrate–carbohydrate interactions in GLYCAM06 than the standard TIP3P water model (Sauter and Grafmüller, 2015; Woods, 2018). Because simulations of AMBER Lipid14 POPC membranes in TIP5P water lead to an unreasonably small area per lipid, we have rescaled the Lennard–Jones interactions between the TIP5P water molecules and the POPC headgroup atoms to obtain the same area per lipid as in standard AMBER Lipid14 simulations with the TIP3P water model (Kav et al., 2020).

Our computational investigation is based on four different simulation systems. We have investigated the *trans*-interaction of a single pair of Le^X^ or Lac 2 glycolipids in a simulation system that consists of a lipid bilayer with one glycolipid anchored in each monolayer (Kav et al., 2020) (see Figure 1A). In this system, the two glycolipids in the different monolayers interact due to the periodic boundary conditions of the simulation box. We have varied the separation of the membrane monolayers by varying the number of water molecules in the simulation box. At each membrane separation, we have generated 10 trajectories with a length of 3 μs for the Le^X^ system and with a length of 1 μs for the Lac 2 system at a temperature of 30°C. The total simulation times at each membrane separation are thus 30 and 10 μs for the Le^X^ and Lac two systems, respectively. The membranes contain in each monolayer 35 POPC lipids besides the single glycolipid and have an area of 23.3 nm^2^. Similarly, we have investigated the *cis*-interaction of a single pair of Le^X^ glycolipids in a simulation system in which two glycolipids are anchored in the same monolayer of the membrane (see Figure 1B). The monolayer with the two Le^X^ glycolipids contains 34 POPC lipids, and the other monolayer contains 36 POPC lipids. We have generated 25 trajectories with a length of 3.6 μs for this system at a temperature of 30°C. The total simulation time for this system is thus 90 µs.

**FIGURE 3:**
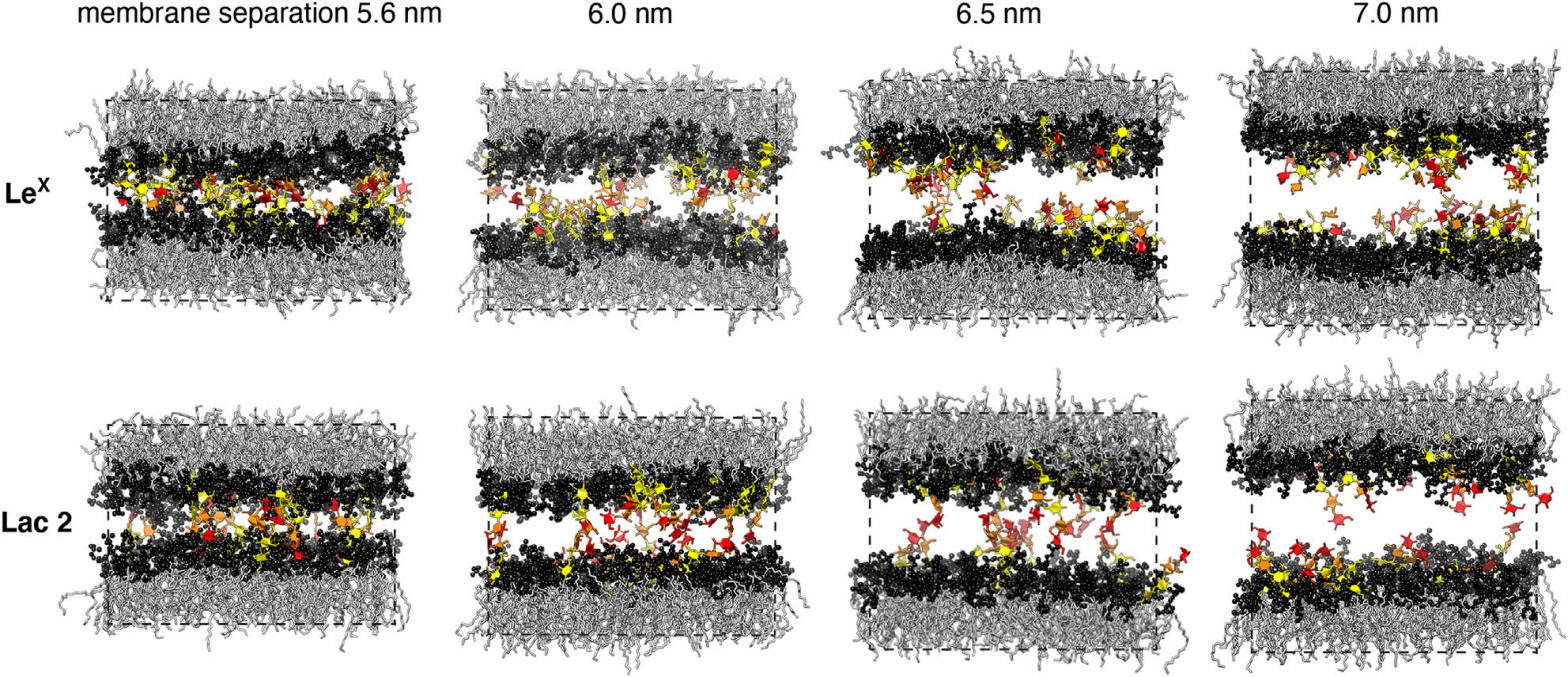
Simulation conformations of membranes with 10 mol% Le^X^ or Lac 2 glycolipids and an area of 63.5 nm^2^ at different membrane separations. The values for the membrane separation correspond to the separation from membrane midplane to membrane midplane and, thus, to the height of the simulation box. The fucose and galactose at the branched tip of the Le^X^ glycolipids and the galactose and glucose at the tip of the Lac 2 glycolipids are represented in red and orange. The remaining monosaccharide units are represented in yellow.

In addition, we have investigated the interplay of *trans*- and *cis*-interactions in a system with 10 Le^X^ or 10 Lac 2 glycolipids in each monolayer of a membrane, besides 90 POPC lipids (see Figure 1C). The fraction of glycolipids in the membrane is thus 10 mol%, and the area of the membrane is 63.5 nm^2^. In this system, the glycolipids *cis*-interact with glycolipids of the same monolayer and *trans*-interact with glycolipids in the other monolayer across the periodic boundaries of the simulation box. By varying the number of water molecules in the simulation box, we have varied the membrane separation (see Figure 3) and have generated 10 trajectories with a length of 1 μs at each separation. The values for the membrane separation in Figure 3 correspond to the separation from membrane midplane to membrane midplane and, thus, to the height of the simulation box. In the simulation systems of Figures 1A,C, the local membrane separation along the membranes is constant and equal to the simulation box height. The local separation of the membranes is not affected by the small membrane shape fluctuations in these systems because the two monolayers of the membrane are coupled in these shape fluctuations.

Finally, we have explored the role of membrane shape fluctuations in glycolipid-mediated adhesion in our largest simulation system, which consists of two membranes with 90 Le^X^ glycolipids and 810 POPC lipids in each of the four monolayers of the membranes (see Figure 1D). In this system, each membrane has an area of 582 nm^2^, and the two aqueous compartments between the membranes both contain 70,776 water molecules so that the average membrane separation is close to the average separation measured in neutron scattering experiments with stacks of membranes that contain 10 mol% of Le^X^ glycolipids (Schneck et al., 2011). The local separation between the two membrane interfaces varies in this system because of the shape fluctuations of the two membranes (see Figure 1D). We have generated 10 trajectories of this system with lengths that vary between 0.95 and 1.12 μs and sum up to a cumulative simulation time of 10.43 µs

We have generated initial structures of the simulation systems in Figures 1A–C by first building POPC lipid membranes using the CHARMM-GUI program (Jo et al., 2008) and subsequent replacement of lipids by Le^X^ or Lac 2 glycolipids, which have the same lipids tails as POPC. After initial minimization and equilibration following standard procedures (Dickson et al., 2014; Kav et al., 2020), we have produced all simulation trajectories in AMBER 16 GPU (Salomon-Ferrer et al., 2013b; Le Grand et al., 2013) at a simulation temperature of 30°C using a Langevin thermostat (Salomon-Ferrer et al., 2013a) with a collision frequency of 5.0 ps^−1^. We have employed a semi-isotropic pressure coupling with a pressure of 1 bar in all directions, which corresponds to a membrane tension of zero, and the Berendsen barostat (Berendsen et al., 1984) with a relaxation time of *τ* = 3 ps for the pressure regulation because of the stability of the semi-isotropic pressure coupling in AMBER 16 GPU with this barostat. We have constrained the bond lengths for hydrogen atoms using the SHAKE algorithm (Ryckaert et al., 1977; Miyamoto and Kollman, 1992) and have set the MD integration timestep to 2 fs. The nonbonded interactions were calculated using the Particle Mesh Ewald (PME) algorithm (Darden et al., 1993; Essmann et al., 1995) for a cutoff length of 1.0 nm.

To generate an initial structure for the large simulation system of Figure 1D, we have used the initial structure for the simulation system of Figure 1C, which contains 10 Le^X^ glycolipids and 90 lipids in each monolayer. We have first replicated this initial structure 9 times to generate the first membrane with 90 Le^X^ glycolipids and 810 lipids in each monolayer and have then duplicated and translated the membrane to obtain the second membrane. In order to increase the computational efficiency, we have applied hydrogen mass repartitioning (Hopkins et al., 2015), which allowed us to increase the MD integration time step to 3 fs, in addition to the equilibration and simulation procedures described above for the smaller systems.

### 2.2 Analysis of *Trans*- and *Cis*-Interactions of Glycolipids

**FIGURE 4:**
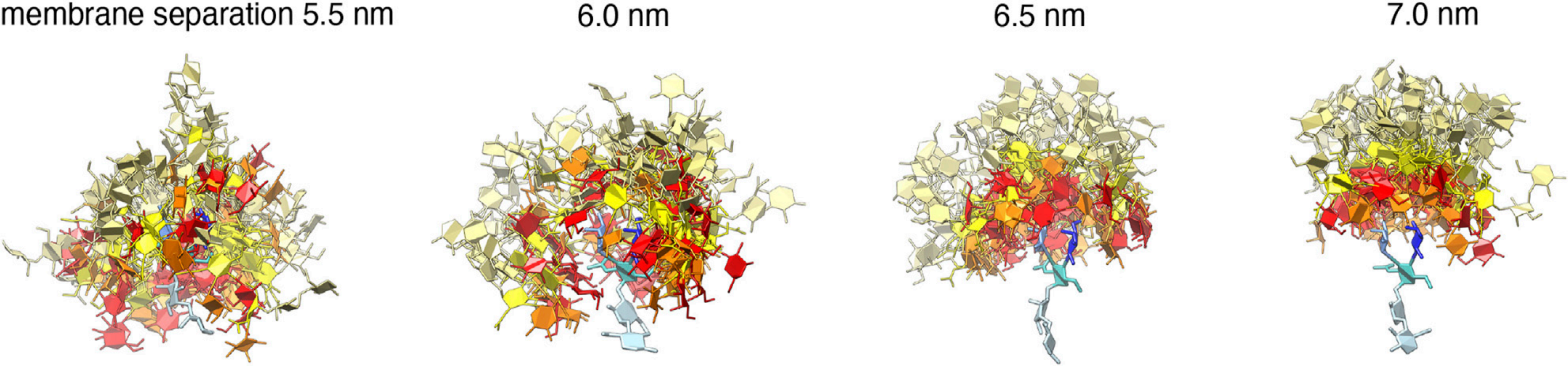
50 randomly selected *trans*-complexes of the carboyhydrate tips of the Le^X^ glycolipids at different membrane separations. The selected complexes exhibit at least 10 contacts between non-hydrogen atoms of the two carbohydate tips and are extracted from the simulation system with a single trans pair shown in Figure 1A. The carbohydrate tip of the lower Le^X^ glycolipid is aligned in the 50 complexes and represented in blue colors, while the carbohydrate tip of the upper glycolipid is represented in red/yellow as in Figure 1.

We have identified interaction events between the carbohydrate tips of the glycolipids along the simulation trajectories as consecutive stretches of simulation frames at intervals of 0.1 ns with nonzero contacts of the tips (Kav et al., 2020). Here, contacts are defined as contacts between non-hydrogen atoms of the two carbohydrate tips within a distance of less than 0.45 nm. The interaction events of the carbohydrate tips can be characterized by their lifetime and by the maximum number of contacts of the events. In an attempt to distinguish between collisions and binding events, only interaction events with a maximum number of contacts that is larger or equal to a cutoff number *n*_*c*_ are considered as binding events. This distinction based on a cutoff number *n*_*c*_ of contacts is somewhat arbitrary because of the fuzzy interactions of the carbohydrates (Kav et al., 2020), which exhibit a large variety of diverse, bound conformations in our simulations, rather than a single binding conformation (see Figure 4). The binding constants determined from our simulations therefore depend—to some extent—on the cutoff number *n*_*c*_. All binding constant values reported here have been calculated for *n*_*c*_ = 5. Binding constant values for *n*_*c*_ = 10 are typically about 10–15% smaller than the values obtained for *n*_*c*_ = 5 (Kav et al., 2020).

We have determined the *trans*-binding constant *K*_trans_ of the two Le^X^ or two Lac 2 glycolipids embedded in the different membrane monolayers of the simulation system shown in Figure 1A as *K*_trans_ = *AP*_*b*_/(1 − *P*_*b*_), where *P*_*b*_ is the probability that the two glycolipid tips are bound, and *A* is the membrane area. The binding probability *P*_*b*_ is simply the fraction of simulation frames belonging to binding events. We have calculated the *trans*-binding probability of the Le^X^ glycolipids from 1,490, 1,609, 588, and 141 binding events observed on our trajectories at the membrane separations 5.5, 6.0, 6.5, and 7.0 nm, respectively. For two Lac 2 glycolipids, we have obtained 609, 413, 183, and 34 such binding events on the trajectories at the corresponding membrane separations. To ensure independence from the initial conformation of the trajectories, we have discarded the first 10% of each trajectory in our calculations of the binding probability *P*_*b*_ of the two molecules. Similarly, we have determined the *cis*-binding constant *K*_cis_ of the two Le^X^ glycolipids embedded in the same membrane monolayer of the simulation system of Figure 1B as *K*_cis_ = *AP*_*b*_/(1 − *P*_*b*_), where *P*_*b*_ is the binding probability obtained from 4,308 binding events on all trajectories, after discarding the first 20% of each trajectory to ensure independence from the initial conformation.

In the simulation system of Figure 1C, a Le^X^ or Lac 2 glycolipid can be involved in several *trans*- and/or *cis*-interactions in a simulation frame. In this system, we have determined the effective *trans*-binding constant as $K_{\text{trans}}=A\;n_{\text{trans}}/{\left(10-n_{\text{trans}}\right)}^{2}$ where *n*_trans_ is the average number of glycolipids in a monolayer engaged in *trans*-interactions. Because each of the monolayers contains 10 glycolipids, the average number of glycolipids that are not engaged in *trans*-interaction is 10 − *n*_trans_. For all our simulations, errors have been calculated as the error of the mean of values obtained for the independent trajectories.

### 2.3 Neutron Scattering Experiments

Experiments were carried out with DPPC membranes doped with 10 mol% glycolipids, whose synthesis has been described elsewhere (Schneider et al., 2001; Schneider et al., 2003; Tanaka et al., 2003; Tanaka et al., 2004). In the glycolipids used in the experiments, the alkyl chains were connected to the glycerol *via* ether bonds, in contrast to the glycolipids in the simulations, where the alkyl chains are connected *via* ester bonds. The Lac 1 glycolipid (Schneider et al., 2001, Schneider et al., 2003; Tanaka et al., 2003; Schneck et al., 2008), Lac 2 glycolipid (Schneider et al., 2001, Schneider et al., 2003; Tanaka et al., 2003), and Gentiobiose glycolipid (Tanaka et al., 2004; Schneck et al., 2008) consist of two saturated hexadecyl chains and of carbohydrate tips connected to the chains *via* a glycerol junction (see Supplementary Figure S1). The Lac 1 and Gentiobiose glycolipids have the disaccharides lactose and gentiobiose as carbohydrate tips, while the Lac 2 glycolipid has a tetrasaccharide tip composed of two lactose units. Neutron diffraction contrast was optimized by using chain-deuterated DPPC and light water (H_2_O) in combination with the Lac 1 and Gentiobiose glycolipids, which were available in their chain-deuterated forms. Consequently, chain-hydrogenous DPPC and heavy water (D_2_O) were used in combination with the Lac 2 glycolipid, which was available in its chain-hydrogenous form. All lipids were initially dissolved in 7:3 mixtures (v/v) of chloroform and methanol at a concentration of 1 mg/ml. Subsequently, mixed solutions with the desired molar ratio were prepared. Of these solutions, 1–2 ml were deposited onto planar Si[100]-substrates with native oxide (Si-Mat, Landsberg/Lech, Germany), which had previously been cut into a rectangular shape (65 mm × 25 mm) and cleaned using a modified RCA method (Kern and Puotinen, 1970). Due to their amphiphilic nature, the lipid mixtures form aligned membrane multilayers on the planar surfaces. To remove residual solvent, the coated wafers were stored at 70°C for 3 h and, subsequently, in a vacuum chamber overnight. At least two heating/cooling cycles between 20 and 80°C were performed at a high relative humidity >95 %.

Neutron diffraction experiments were performed on the high-resolution diffractometer D16 at the Institut Laue-Langevin (ILL, Grenoble, France). The incident beam with a wavelength of *λ* = 0.474 nm (Δ*λ*/*λ* ≈ 1%) reached the sample plane through the aluminum windows of the sample chamber, with an adjustable angle of incidence Ω. Scattering occurs into various directions at angles Γ with respect to the incident beam. For each Ω, the Γ-dependent intensity is recorded using a position-sensitive ^3^He detector with 128 × 128 channels and a spatial resolution of 2 mm. By rotating the sample stage and, thus, by stepwise variation of Ω, two-dimensional maps of the intensity as a function of Γ and Ω were recorded (see images in Supplementary Figure S2). During this procedure, the intensity was normalized to the intensity of the incident beam (*via* an in-beam monitor), the pixel sensitivity and solid angle, and the illuminated sample area. Bragg peaks associated with the lamellar period *D* of the membrane multilayers are found where the specular condition (Γ = 2Ω) coincides with the Bragg condition (Γ = 2 arcsin(*nλ*/(2*D*)), see plots in Supplementary Figure S2), where n∈N . The average membrane separation is identical to the lamellar period and obtained by solving the Bragg condition for *D*.

For the diffraction experiments, a liquid cell designed for solid-supported membrane multilayers (Schneck et al., 2008, Schneck et al., 2009) was used: two planar Si substrates, one of them coated with the membrane multilayers, were assembled into a sandwich-like configuration with small glass pieces (thickness: 0.10 mm) as spacers between them. The space between the two wafers was then filled with H_2_O- or D_2_O-based aqueous solutions containing 100 mM NaCl and 5 mM Hepes (Fluka, Taufkirchen, Germany) and, optionally, 5 mM CaCl_2_. During the diffraction experiments, the measurement cell was placed in a climate chamber at controlled temperature and high relative humidity ( >95 %) to minimize water evaporation. Temperature was kept at 60°C such that the bilayers were in the fluid L_*α*_-phase in all cases.

## 3 Results

### 3.1 *Trans*-Interactions of Glycolipids in Simulations

**FIGURE 5:**
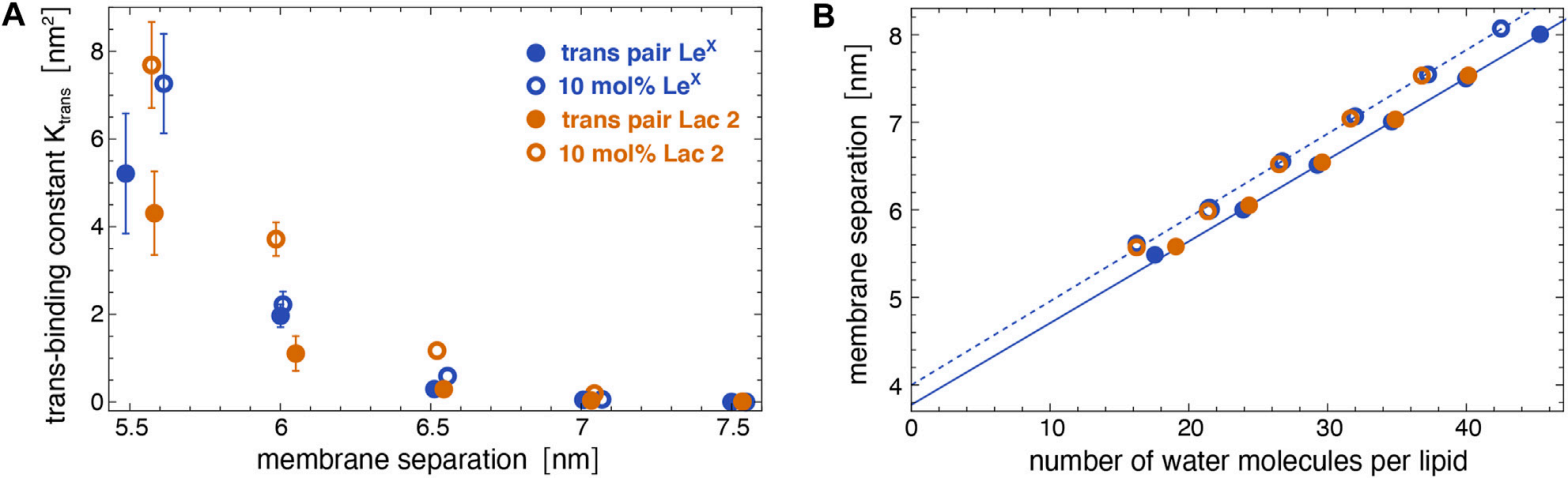
**(A)** *Trans*-binding constants of Le^X^ and Lac 2 glycolipids obtained in the membrane system of Figure 1A with a single *trans*-pair of glycolipids (full circles) and in the system of Figure 1C with 10 mol% glycolipids (open circles) at different membrane separations. **(B)** Membrane separation *versus* number of water molecules per lipid in the simulation box for the same systems as in subfigure A. The membrane separation from membrane midplane to midplane corresponds to the height of the simulation box with periodic boundaries.

Figure 5A illustrates the *trans*-binding constant *K*_trans_ of Le^X^ glycolipids and of Lac 2 glycolipids obtained in the simulation system of Figure 1A with a single *trans*-pair of glycolipids and in the system of Figure 1C with 10 mol% glycolipids. In both systems, the glycolipids in the different monolayers *trans*-interact due to the periodic boundary conditions of the simulation box. In the system of Figure 1C, glycolipids in the same monolayer *cis*-interact in addition to the *trans*-interactions with glycolipids in the apposing monolayer. We have determined *K*_trans_ at different membrane separations by varying the number of water molecules in the simulation box (see Figure 5B). The values for the membrane separation *l* given in Figure 5 correspond to the separation from membrane midplane to membrane midplane and, thus, to the height of the simulation box. At the smallest separation (*l* = 5.5–5.6 nm) considered in the different simulation systems, the number of water molecules per lipid in the simulation box ranges from 16 to 19, depending on the system. Simulation conformations of the membranes with 10 mol% Le^X^ glycolipids or Lac 2 glycolipids at the different separations are shown in Figure 3.

The *trans*-binding constants *K*_trans_ of the glycolipids strongly decrease with increasing membrane separation. This decrease in *trans*-interactions can be understood from a decrease in the overlap of the carbohydrate tips of the glyoclipids. Figure 4 illustrates 50 randomly selected *trans*-complexes of the Le^X^ glycolipid tips with at least 10 contacts of non-hydrogen atoms at each of the membrane separations *l* = 5.5, 6.0, 6.5, and 7.0 nm. The carbohydrate tip of the lower Le^X^ glycolipid is aligned in the 50 complexes and represented in blue colors, while the carbohydrate tip of the upper glycolipid is represented in red/yellow colors. The clouds of red/yellow carbohydrates illustrate that the interactions of the glycolipids are fuzzy, that is, the carbohydrate tips exhibit a large variety of bound conformations in our simulations. The overlap of the cloud of the upper, red/yellow carbohydrates with the lower, blue carbohydrate decreases with increasing membrane separation. At the membrane separation *l* = 5.5 nm, the Le^X^ glycolipids interact *via* their entire carbohydrate tips. At *l* = 6.0 nm, the interactions are limited to the branched trisaccharide of the glycolipid tip, and at *l* = 6.5 and 7.0 nm, the interactions are further restricted to the galactose and fucose monosaccharides at the branched end of the Le^X^ glycolipid.

A comparison of the *K*_trans_ values from the two simulation systems indicates that *cis*-interactions tend to strengthen the *trans*-interactions. At a given membrane separation, the *K*_trans_ values obtained in the system with 10 mol% glycolipids, in which the glycolipids can also *cis*-interact, are slightly larger than the *K*_trans_ values obtained in the system with a single *trans*-pair of glycolipids. For Le^X^ glycolipids, we have determined the *cis*-binding constant *K*_cis_ = 7.9 ± 1.2 nm^2^ in the simulation system of Figure 1C with a single *cis*-pair. This value of *K*_cis_ is comparable to and even slightly larger than the *K*_trans_ values at the membrane separation 5.5 nm, at which the Le^X^ glycolipids can *trans*-interact with their entire carbohydrate tips according to Figure 4. The comparable magnitude of *cis*- and *trans*-interactions at this membrane separation is also reflected by the average number of Le^X^ glyolipids engaged in *trans*- and *cis*-interactions in the membrane system with 10 mol% of glycolipids. We find that, on average, about four out of the 10 Le^X^ glyolipids of a monolayer are engaged in *trans*-interactions, while about three of the 10 Le^X^ glyolipids are engaged in *cis*-interactions at the smallest membrane separation considered in our simulations. Because of the fuzzy binding, *trans*- and *cis*-interactions of the glycolipids are not mutually exclusive, and trimer and higher multimers in different *trans*- and *cis*-combinations frequently occur along the simulation trajectories.

In addition to Le^X^ and Lac 2 glycolipids, we have also determined the *trans*-binding constant *K*_trans_ of Lac 1 glycolipids with a small disaccharide galactose–glucose tip in the system of Figure 1C at the membrane separations *l* = 5.3, 5.6, 6.0, and 6.5 nm. The carbohydrate tip of the Lac 1 glycolipid thus has half the size of the tetrasaccharide tip of the Lac 2 glycolipid. For these Lac 1 glycolipids, we only obtain noticeable *trans*-interactions at the smallest membrane separation 5.3 nm with *K*_trans_ = 0.90 ± 0.07 nm^2^. This value of *K*_trans_ is comparable to the *K*_trans_ value of Lac 2 glycolipids at the separation 6.5 nm in the same system with 10 mol% of glycolipids (see Figure 5A). At the membrane separation 5.6 nm, the *K*_trans_ value of the Lac 1 glycolipids is already strongly reduced to 0.06 ± 0.01 nm^2^ and vanishingly small at the separations 6.0 and 6.5 nm.

### 3.2 Average Separations of Membranes With Glycolipids From Neutron Scattering Experiments

**FIGURE 6:**
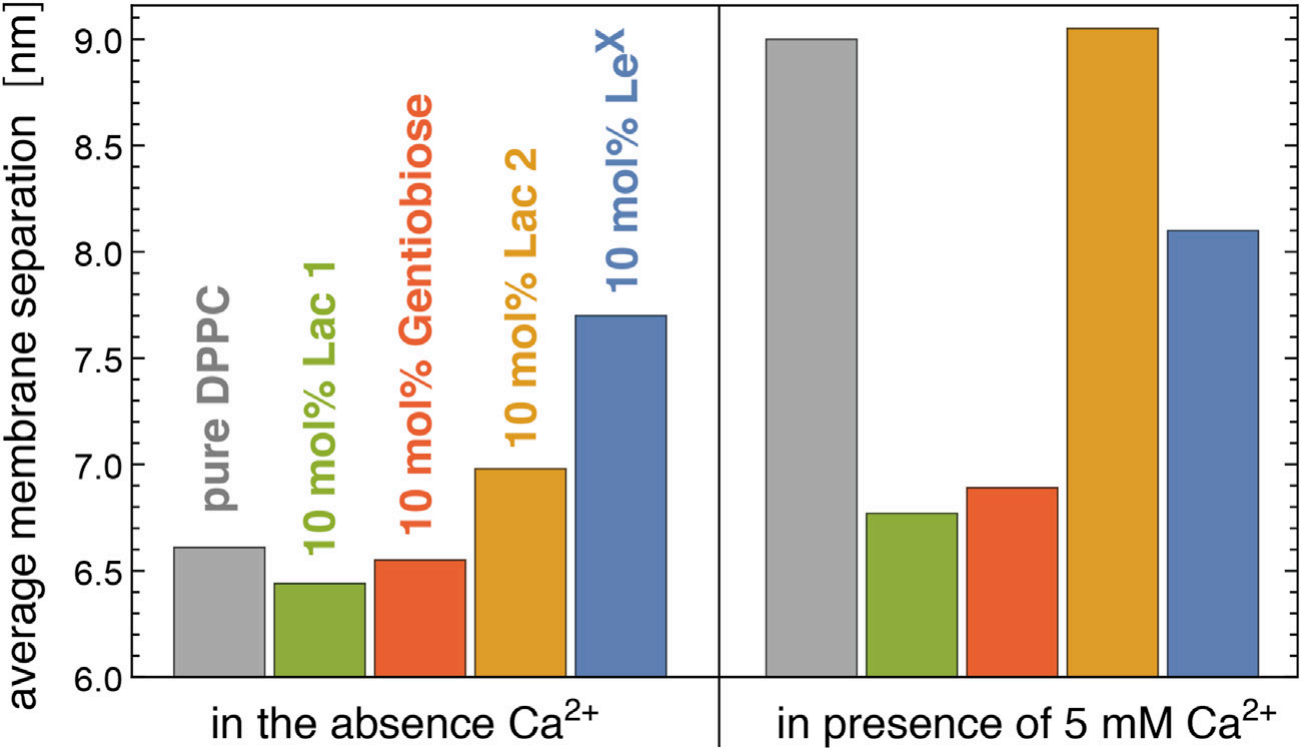
Average membrane separation in stacks of pure DPPC membranes and of DPPC membranes with 10 mol% Lac 1, Gentiobiose, Lac 2, and Le^X^ glycolipids measured by neutron scattering in the absence and presence of Ca^2+^. The average membrane separations of Le^X^ glycolipids are from the study by Schneck et al. (2011).

Figure 6 illustrates neutron scattering results for the average separation in stacks of DPPC membranes that contain 10 mol% of Lac 1, Gentiobiose, Lac 2, or Le^X^ glycolipids. The neutron scattering experiments have been performed at a temperature of 60°C at which the DPPC membrane is fluid. We find that the average separation between adjacent membranes in the stack strongly depends on the glycolipid embedded in the membranes. In the absence of Ca^2+^ ions that associate with PC lipid headgroups and induce an electrostatic repulsion of the membranes (Lis et al., 1981; Altenbach and Seelig, 1984), the average membrane separation of DPPC membranes that contain 10 mol% of Lac 1 or Gentiobiose lipid with small disaccharide tips is close to the average separation of pure DPPC membranes. The average separations of membranes with 10 mol% of Le^X^ and Lac 2 glycolipids, in contrast, are considerably larger than the average separation of pure DPPC membranes. In the presence of 5 mM Ca^2+^ ions, the average separation of pure DPPC membranes strongly increases by 2.4 nm, whereas the average separation of membranes with 10 mol% of Lac 1, Gentiobiose, and Le^X^ glycolipids only increases by about 0.3–0.4 nm, compared to the separation in the absence of Ca^2+^. Interestingly, the average separation of membranes with 10 mol% of Lac 2 glycolipids increases to a value close to the separation of pure DPPC membranes in the presence of 5 mM Ca^2+^.

For Le^X^ glycolipids, the average membrane separation from neutron scattering has been previously reported at concentrations of 0 mol% (pure DPPC), 2 mol%, 5 mol%, 10 mol%, and 25 mol% both in the absence of Ca^2+^ and the presence of 1, 2, and 5 mM Ca^2+^ (Schneck et al., 2011). At the different Ca^2+^ concentrations, the average separation of the membranes attains values that are rather similar at large concentrations of the Le^X^ glycolipids, which indicates that these average separations result from stable membrane adhesion mediated by the Le^X^ glycolipids. The average membrane separations are significantly larger than the separations at which Le^X^ glycolipids *trans*-interact in our simulations. Membrane shape fluctuations therefore seem to be important to bring the glycolipids in the apposing membrane surfaces into local contact.

### 3.3 Interplay of Trans-Binding and Membrane Shape Fluctuations in Simulations

We have investigated the role of membrane shape fluctuations in our largest simulation system that consists of two membranes with 10 mol% of Le^X^ glycolipids and an area of 24.1 × 24.1 nm^2^. In this system, we have adjusted the average separation from membrane midplane to midplane to 7.7 nm, the average membrane separation measured in the neutron scattering experiments. Using the same separation value is justified because fluid DPPC membranes (Lis et al., 1982) and the POPC membranes simulated here have virtually the same thickness when the water layer thickness is defined consistently through the water volume and the area per lipid, as described in the study by Kanduc et al. (2017). In our simulations, the two aqueous compartments between the membranes contain the same number of water molecules, and the simulation box height of 15.4 nm is twice as large as the average membrane separation because we have two membranes. The membrane shape fluctuations can be quantified by the relative roughness of the two membranes, which is the standard deviation $\xi_{⊥}=\sqrt{\left\langle {\left(l-\bar{l}\right)}^{2}\right\rangle }$ of the local separation *l* of the membranes from the average separation $\bar{l}$ . To calculate the roughness *ξ*_⊥_ of the membranes in a simulation conformation, we have divided the *x*-*y*-plane of our simulation box, which is, on average, parallel to the membranes, into 16 × 16 quadratic patches. This discretization of the *x*-*y*-plane leads to 16 × 16 membrane patches in each of the two membranes. The membrane patches have an area of about 1.5 × 1.5 nm^2^ and contain, on average, 3.5 POPC lipids or glycolipids in each monolayer. For each patch in each of the two membranes, we have determined the *z*-coordinate of the center of mass of the lipid tails in the same monolayer and have calculated the *z*-position of the membrane midplane as the average value of the *z*-coordinates for the monolayers of the membrane patch. From the two *z*-positions of the two apposing membrane patches with the same *x*-*y*-position, we have obtained two values for the local separation *l* between these membrane patches, which add up to the simulation box height.

**FIGURE 7:**
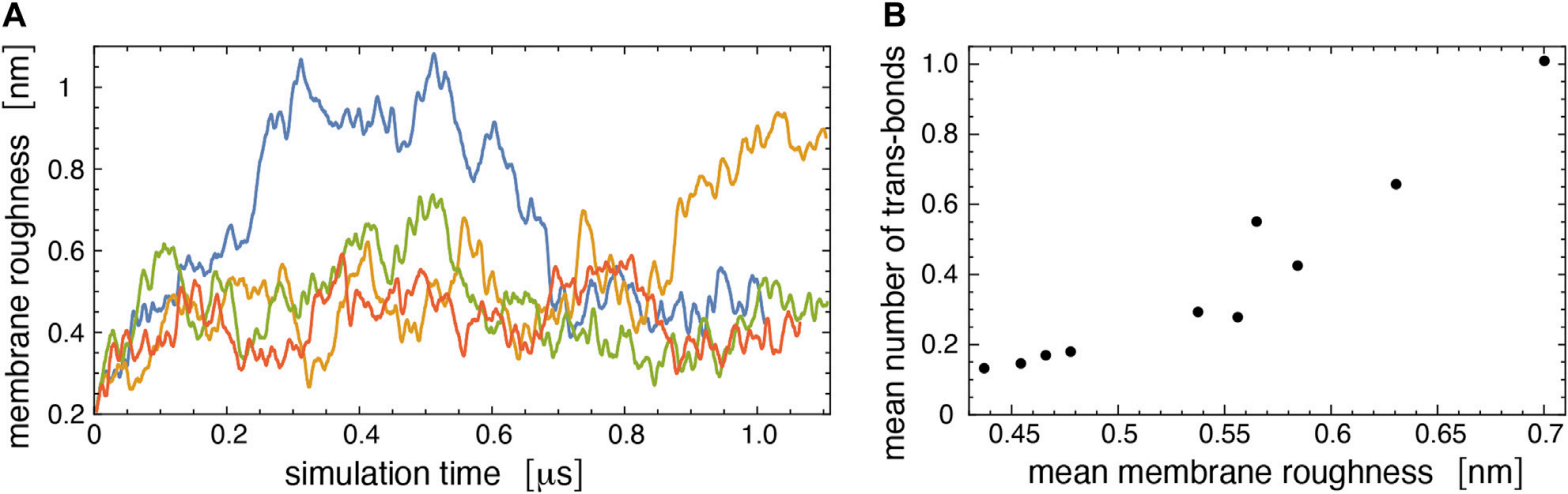
**(A)** Membrane roughness along four simulation trajectories of our large simulation system. The roughness value at a given simulation time is averaged (“smoothened”) over 50 values for simulation frames at intervals of 0.15 ns around the time point. The final conformations of the four trajectories are shown in Figure 8. The conformation at the top right of Figure 8 with large roughness is the last conformation of the trajectory with roughness values shown in yellow. **(B)** Mean number of *trans*-bonds *versus* mean membrane roughness for the 10 trajectories of our large simulation systems. The mean values have been calculated after discarding the first 150 ns of the trajectories as relaxation time.

**FIGURE 8:**
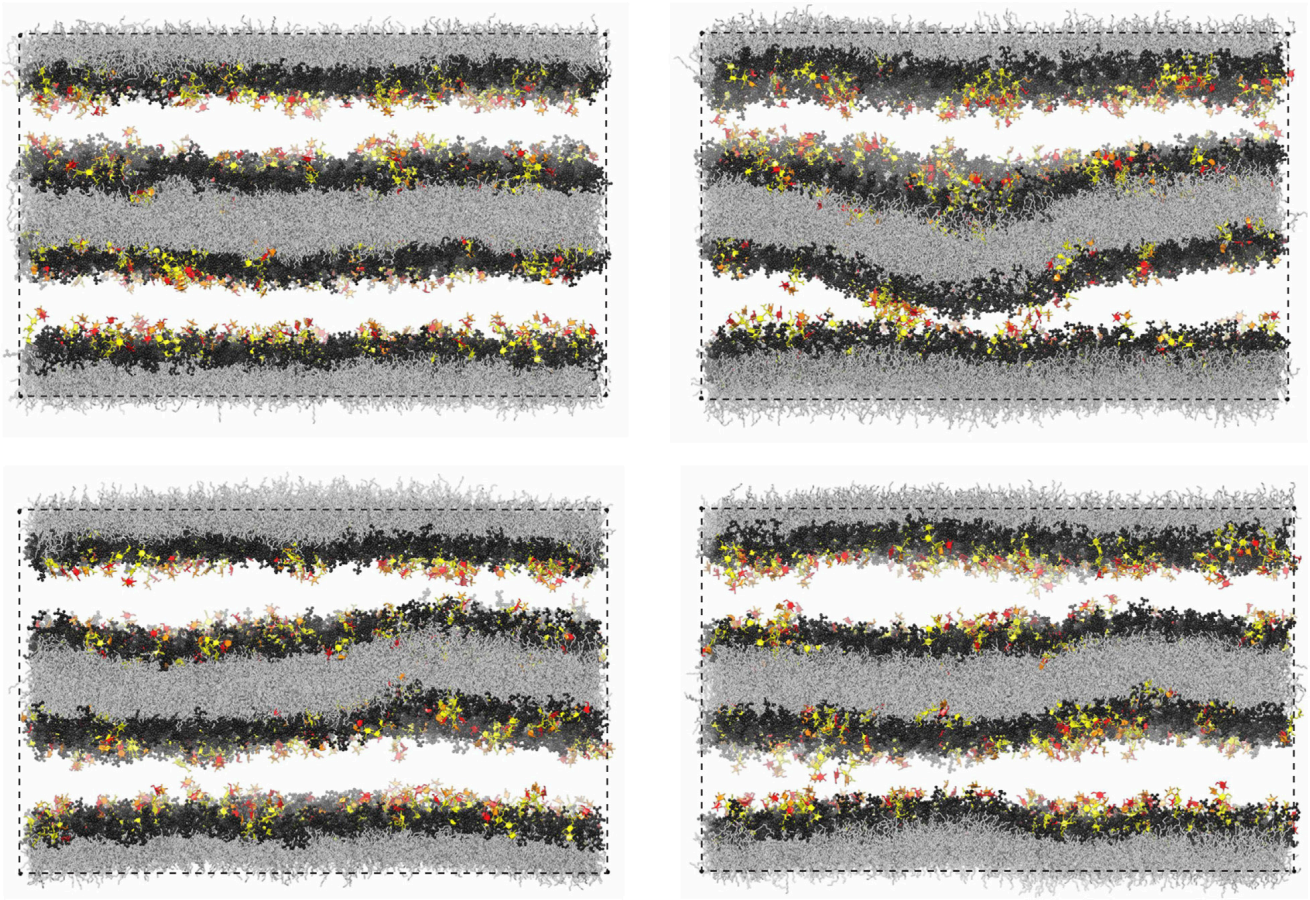
Final simulation conformations on four out of the 10 simulation trajectories of our large simulation system with two membranes. The final conformation on a 5th trajectory is shown in Figure 1D.

Figure 7A illustrates the membrane roughness along four of the 10 simulation trajectories for this system. Depending on the trajectory, the membrane roughness varies between about 0.3 and 1 nm, after an initial relaxation of about 0.15 μs, during which the roughness increases on all trajectories. The last conformations of these four trajectories are shown in Figure 8. The roughness of the conformation at the top right of Figure 8, which corresponds to the yellow trajectory in Figure 7A, is visibly larger than the roughness of the other three conformations and leads to *trans*-interactions of Le^X^ glycolipids in the lower membrane interface. The interplay of membrane roughness and *trans*-interactions is also supported by the correlation between the mean membrane roughness and the mean number of *trans*-bonds of the 10 trajectories after the initial relaxation of about 0.15 µs (see Figure 7B).

**FIGURE 9:**
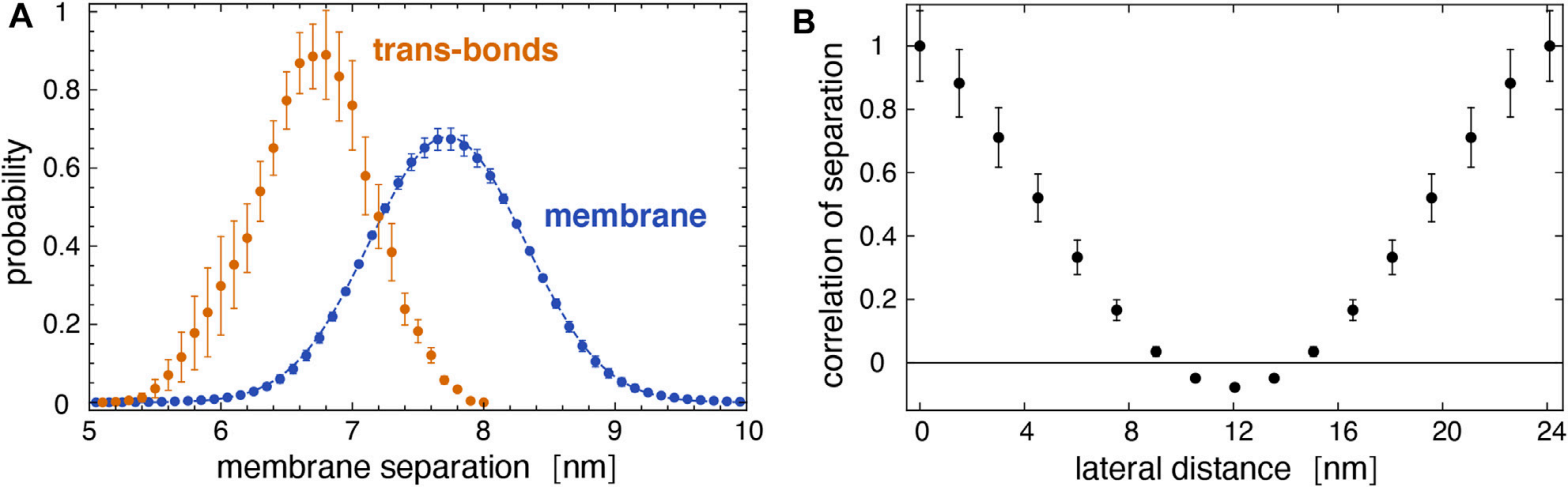
**(A)** Distribution of local separations for the entire membrane (blue) and distribution of separations for membrane sites with trans bonds (yellow) obtained from the 10 trajectories of our large membrane system. The dashed blue line represents a fit of the Gaussian function $P\left(l\right)≃\exp\left[-{\left(l-\bar{l}\right)}^{2}/2\xi_{⊥}^{2}\right]/\left(\sqrt{2\pi }\xi_{⊥}\right)$ with average separation $\bar{l}=7.72$ nm and relative membrane roughness *ξ*_⊥_ = 0.53 nm. **(B)** Spatial correlation of the local membrane separation along the lateral distance parallel to the *x*- and *y*-axis of the simulation box. The errors in this figure are estimated as the error of the mean of the probability distributions and correlation functions for the individual trajectories.

As expected from Figure 5, *trans*-bonds of Le^X^ glycolipids along the trajectories only occur at local membrane separations *l* that are smaller than the average separation $\bar{l}=7.7$ nm of the membranes (see Figure 9A). The distribution of the local membrane separations for membrane patches with *trans*-bound Le^X^ glycolipids is centered around 6.7 nm, while the distribution for all membrane patches is centered at the average membrane separation of 7.7 nm of the membranes. The standard deviation of the distribution *P*(*l*) is the membrane roughness *ξ*_⊥_ = 0.53 nm averaged over all trajectories. This average membrane roughness along our trajectories is somewhat smaller than the membrane roughness *ξ*_⊥_ = 0.73 ± 0.03 nm obtained from neutron scattering experiments (Schneck et al., 2011; Kav et al., 2020), likely because the membrane area in our large simulation system is still somewhat too small to allow all relevant fluctuation modes of the membranes. The lateral correlation function of the local separation in Figure 9B indicates remaining correlations between membrane patches with the maximum distance of 12 nm along the *x*- and *y*-axis of the simulation box, which lead to negative values of the correlation function at this maximum distance.

## 4 Discussions and Conclusion

Our simulations and neutron scattering results provide complementary information on glycolipid interactions in membrane adhesion. The neutron scattering experiments inform on the average membrane separation and thermal membrane roughness in stacks of membranes with glycolipids. In contrast, the simulations provide a molecular view of the interactions between the carbohydrate tips of the glycolipids, at different, fixed local membrane separations in the simulation systems of Figures 1A,C and at a fixed average membrane separation of 7.7 nm in the large system with two membranes of Figure 1D. In our simulations of this large system, the shape fluctuations of the membranes lead to local membrane separations smaller than the average separation of the membranes, which enables *trans*-binding. The average membrane separation obtained from the neutron scattering experiments likely results from a balance between the glycolipid-mediated attraction of the membranes and the steric repulsion of the fluctuating membranes. In the absence of Ca^2+^, the average separation of membranes with 10 mol% Lac 2 glycolipids is smaller than the average separation of membranes with 10 mol% Le^X^ glycolipids (see Figure 6). In principle, this smaller separation for Lac 2 glycolipids may result from a stronger glycolipid-mediated attraction or from a reduced steric membrane repulsion, or both. The carbohydrate tips of the Le^X^ and Lac 2 glycolipids have the same length. A structural difference is that the carbohydrate tip of the Le^X^ glycolipid is branched and, thus, somewhat bulkier at its end (see Figure 2), which may lead to an increased steric repulsion caused by Le^X^ glycolipids. An increased glycolipid-mediated attraction of Lac 2 glycolipids is supported by the *trans*-binding constants *K*_trans_ obtained from our simulations (see Figure 5). At the membrane separations 6.0 and 6.5 nm, the *K*_trans_ values of Lac 2 glycolipids are significantly larger than the values of the Le^X^ glycolipids.

The interplay of membrane shape fluctuations and glycolipid *trans*-binding observed in our largest membrane system illustrates that *trans*-binding of Le^X^ glycolipids is possible at the large average membrane separation obtained from the neutron scattering experiments. A caveat is that only a tiny fraction of the Le^X^ glycolipids is engaged in *trans*-interactions in our simulations. The membranes in our largest simulation system contain 90 Le^X^ glycolipids in each monolayer and, thus, 360 Le^X^ glycolipids in total, but the average number of *trans*-bonds for both membrane interfaces in this system only ranges from 0.15 to 1 along the 10 simulation trajectories, depending on the average membrane roughness of the trajectory (see Figure 7B). Even along the simulation trajectory with the largest average roughness of *ξ*_⊥_≃ 0.7 nm that agrees with the roughness *ξ*_⊥_ = 0.73 ± 0.03 nm obtained from the neutron scattering experiments (Schneck et al., 2011; Kav et al., 2020), on average, only about 1 Le^X^ glycolipid *trans*-bond occurs in the simulation system, together on both membrane interfaces of the system. Membrane shape fluctuations on length scales somewhat larger than those accessible in our simulation system may lead to more pronouced *trans*-binding. But overall, the *trans*-interactions of Le^X^ glycolipids in our simulations at the average membrane separation observed in the neutron scattering experiments appear too weak for glycolipid-mediated membrane adhesion, although adhesion has previously been postulated to only require very few *trans*-bonds of Le^X^ glycolipids (Schneck et al., 2011). Of course, there are important differences in the MD simulations and neutron scattering experiments. The neutron scattering experiments were conducted at a temperature of 60°C at which the DPPC membrane is fluid. Because the MD force fields are calibrated at lower temperatures, we chose POPC membranes for the simulations, which are also fluid at the simulation temperature of 30°C. However, as noted before, both membranes have very similar thicknesses in the fluid phase. Another difference, as noted before as well, is that the alkyl chains of the glycolipids are ether-bonded to the glycerol moiety in the experiments but ester-bonded in the simulations. The ensuing difference in the hydrophobicity of this connection may lead to differences in the anchoring depth of the glycolipid, but likely to a very small extent.

For membranes with the smaller Lac 1 and Gentiobiose glycolipids, which have disaccharides as carbohydrate tips, the average separation is close to the average separation of pure DPPC membranes in the absence of Ca^2+^ (see Figure 6). In the presence of 5 mM Ca^2+^, in contrast, the average separation of pure DPPC membranes strongly increases, whereas the average separation of membranes with Lac 1 and Gentiobiose glycolipids increases rather weakly. These results appear to indicate that the *trans*-interactions of the Lac 1 and Gentiobiose glycolipids strengthen the binding minimum of pure DPPC membranes, which is dominated by the interplay of van der Waals attraction and the short-ranged hydration repulsion (Lis et al., 1982; Kanduc et al., 2017), against electrostatic repulsion induced by Ca^2+^. It is important to note that membrane shape fluctuations are also essential for the *trans*-interactions of these small glycolipids because the local separations at which these *trans*-interactions can occur in our simulations are still significantly smaller than the average separations of the membranes in the neutron scattering experiments. For Lac 1 glycolipids, we only obtain noticeable *trans*-interactions at membrane separations *l* = 5.3 nm in our simulations and strongly reduced or vanishing *trans*-interactions at separations of 5.6 nm and larger (see Section 3.1).

Interestingly, the average separation of membranes with Lac 2 glycolipids strongly increases in the presence of 5 mM Ca^2+^, similarly to pure DPPC membranes (see Figure 6). In contrast, the average separation of membranes with Le^X^ glycolipids increases only moderately in the presence of Ca^2+^, similarly to membranes with Lac 1 and Gentiobiose glycolipids. At the larger average separation of the membranes with Le^X^ and Lac 2 glycolipids, the van der Waals attraction of the DPPC lipids is much weaker and, thus, likely negligible in the interplay between the *trans*-interactions of the glycolipids and membrane shape fluctuations. As discussed above, the smaller average separation of membranes with Lac 2 glycolipids in the absence of Ca^2+^ points toward stronger *trans*-interactions of the Lac glycolipids than those of Le^X^ glycolipids. Such stronger *trans*-interactions are also supported by the larger *trans*-binding constants *K*_trans_ of Lac 2 glycolipids obtained from our simulations at the membrane separations 6.0 and 6.5 nm for a glycolipid concentration of 10 mol% as in the experiments (see Figure 5). Therefore, the strong increase in the average separation of membranes with Lac 2 glycolipids in the presence of 5 mM Ca^2+^ remains puzzling. One possible explanation is that the *trans*-interactions of Le^X^ glycolipids are strengthened by Ca^2+^. Indeed, several groups have reported that Le^X^ binding strongly depends on Ca^2+^ (Geyer et al., 2000; de la Fuente et al., 2001; Hernáiz and de la Fuente, 2002; Gourier et al., 2005; Nodet et al., 2007; Kunze et al., 2013; Witt et al., 2016), whereas one group has observed no dependence on Ca^2+^ in atomic force microscopy experiments of Le^X^ unbinding (Tromas et al., 2001). As pointed out by Kunze et al. (2013), the Ca^2+^ concentration used by most of these groups are of the order of 10 mM, which is far beyond physiological Ca^2+^ concentrations but comparable to the Ca^2+^ concentration of 5 mM in our neutron scattering experiments. In vesicle adhesion experiments, Kunze et al. (2013) observed a rather small increase in the number of bound vesicles for a Ca^2+^ concentration of 0.9 mM, compared to experiments in the absence of Ca^2+^. However, a strong increase in the number of bound vesicles in the experiments occurred for a Ca^2+^ concentration of 10 mM. Another, more speculative explanation is that the interplay of the short-ranged *trans*-interactions of Lac 2 glycolipids with the longer-ranged electrostatic repulsion induced by Ca^2+^ leads to a lateral segregation (Weikl et al., 2002a; Weikl et al., 2002b) in membranes with Lac 2 glycolipids but not in membranes with Le^X^ glycolipids. Such a lateral segregation into 1) membrane domains with larger glycolipid concentration and smaller membrane separation and 2) membrane domains with smaller glycolipid concentration and larger membrane separation can lead to overall larger average separations and depends on the strength and range of the *trans*-interactions and the strength of the *cis*-interactions between the glycolipids. The smaller average separation of membranes with Lac 2 glycolipids in the absence of Ca^2+^ may make these membranes more prone to lateral segregation in the interplay with electrostatic repulsion than membranes with Le^X^ glycolipids.

A challenging goal for future simulations is to determine the equilibrium separation of the membranes that results from the interplay of attractive interactions and steric membrane repulsion. In our simulations, the average separation of the membranes is constrained by the number of water molecules between the membranes. Such future simulations with variable average membrane separation are challenging because they either require water exchange between the aqueous compartments of simulations with explicit water or implicit water simulations with reliable force fields for lipid membranes and glycolipids. Such force fields likely need to be atomistic because of inherent limitations of coarse-grained force fields in capturing binding affinities (Robustelli et al., 2018). A further challenge is the expectable slow relaxation of the average membrane separation in simulations without constraints on this separation, which requires long simulation times.

## Data Availability

The raw data supporting the conclusion of this article will be made available by the authors, without undue reservation.

## References

[B1] Altenbach C. Seelig J. (1984). Calcium Binding to Phosphatidylcholine Bilayers as Studied by Deuterium Magnetic Resonance. Evidence for the Formation of a Calcium Complex with Two Phospholipid Molecules. Biochemistry 23, 3913–3920. 10.1021/bi00312a019 6487586

[B2] Arnaud J. Audfray A. Imberty A. (2013). Binding Sugars: from Natural Lectins to Synthetic Receptors and Engineered Neolectins. Chem. Soc. Rev. 42, 4798–4813. 10.1039/c2cs35435g 23353569

[B3] Berendsen H. J. C. Postma J. P. M. van Gunsteren W. F. DiNola A. Haak J. R. (1984). Molecular Dynamics with Coupling to an External bath. J. Chem. Phys. 81, 3684–3690. 10.1063/1.448118

[B4] Boudière L. Michaud M. Petroutsos D. Rébeillé F. Falconet D. Bastien O. (2014). Glycerolipids in Photosynthesis: Composition, Synthesis and Trafficking. Biochim. Biophys. Acta (Bba) - Bioenerg. 1837, 470–480. 10.1016/j.bbabio.2013.09.007 24051056

[B5] Bucior I. Scheuring S. Engel A. Burger M. M. (2004). Carbohydrate-carbohydrate Interaction Provides Adhesion Force and Specificity for Cellular Recognition. J. Cel Biol. 165, 529–537. 10.1083/jcb.200309005 PMC2172358 15148309

[B6] Darden T. York D. Pedersen L. (1993). Particle Mesh Ewald: AnN⋅Log(N) Method for Ewald Sums in Large Systems. J. Chem. Phys. 98, 10089–10092. 10.1063/1.464397

[B7] Day C. J. Tran E. N. Semchenko E. A. Tram G. Hartley-Tassell L. E. Ng P. S. K. (2015). Glycan:glycan Interactions: High Affinity Biomolecular Interactions that Can Mediate Binding of Pathogenic Bacteria to Host Cells. Proc. Natl. Acad. Sci. USA 112, E7266–E7275. 10.1073/pnas.1421082112 26676578 PMC4702957

[B8] de la Fuente J. M. Barrientos A. G. Rojas T. C. Rojo J. Cañada J. Fernández A. (2001). Gold Glyconanoparticles as Water-Soluble Polyvalent Models to Study Carbohydrate Interactions. Angew. Chem. Int. Ed. 40, 2257–2261. 10.1002/1521-3773(20010618)40:12<2257:aid-anie2257>3.0.co;2-s 29711834

[B9] de la Fuente J. M. Eaton P. Barrientos A. G. Menéndez M. Penadés S. (2005). Thermodynamic Evidence for Ca^2+^-Mediated Self-Aggregation of Lewis X Gold Glyconanoparticles. A Model for Cell Adhesion via Carbohydrate−Carbohydrate Interaction. J. Am. Chem. Soc. 127, 6192–6197. 10.1021/ja0431354 15853323

[B10] Dickson C. J. Madej B. D. Skjevik Å. A. Betz R. M. Teigen K. Gould I. R. (2014). Lipid14: The Amber Lipid Force Field. J. Chem. Theor. Comput. 10, 865–879. 10.1021/ct4010307 PMC3985482 24803855

[B11] Essmann U. Perera L. Berkowitz M. L. Darden T. Lee H. Pedersen L. G. (1995). A Smooth Particle Mesh Ewald Method. J. Chem. Phys. 103, 8577–8593. 10.1063/1.470117

[B12] Geyer A. Gege C. Schmidt R. R. (2000). Calcium-Dependent Carbohydrate-Carbohydrate Recognition between Lewis X Blood Group Antigens. Angew. Chem. Int. Ed. 39, 3245–3249. 10.1002/1521-3773(20000915)39:18<3245:aid-anie3245>3.0.co;2-9 11028065

[B13] Gourier C. Pincet F. Perez E. Zhang Y. Zhu Z. Mallet J.-M. (2005). The Natural Lewis X-Bearing Lipids Promote Membrane Adhesion: Influence of Ceramide on Carbohydrate-Carbohydrate Recognition. Angew. Chem. Int. Edition 44, 1683–1687. 10.1002/anie.200461224 15693050

[B14] Hernáiz M. J. de la Fuente J. M. Barrientos Á. G. Penadés S. Barrientos Á. G. Penadés S. (2002). A Model System Mimicking Glycosphingolipid Clusters to Quantify Carbohydrate Self-Interactions by Surface Plasmon Resonance. Angew. Chem. Int. Ed. 41, 1554–1557. 10.1002/1521-3773(20020503)41:9<1554:aid-anie1554>3.0.co;2-3 19750663

[B15] Hopkins C. W. Le Grand S. Walker R. C. Roitberg A. E. (2015). Long-time-step Molecular Dynamics through Hydrogen Mass Repartitioning. J. Chem. Theor. Comput. 11, 1864–1874. 10.1021/ct5010406 26574392

[B16] Jo S. Kim T. Iyer V. G. Im W. (2008). CHARMM-GUI: a Web-Based Graphical User Interface for CHARMM. J. Comput. Chem. 29, 1859–1865. 10.1002/jcc.20945 18351591

[B17] Kanduč M. Schlaich A. de Vries A. H. Maréchal E. Demé B. Netz R. R. (2017). Tight Cohesion between Glycolipid Membranes Results from Balanced Water-Headgroup Interactions. Nat. Commun. 8, 14899. 10.1038/ncomms14899 28367975 PMC5382269

[B18] Kav B. (2019). Membrane Adhesion Mediated via Lipid-Anchored Saccharides. Potsdam, Germany: Universität Potsdam. Doctoral thesis. 10.25932/publishup-42879

[B19] Kav B. Grafmüller A. Schneck E. Weikl T. R. (2020). Weak Carbohydrate-Carbohydrate Interactions in Membrane Adhesion Are Fuzzy and Generic. Nanoscale 12, 17342–17353. 10.1039/d0nr03696j 32789381

[B20] Kern W. Puotinen D. A. (1970). Cleaning Solutions Based on Hydrogen Peroxide for Use in Silicon Semiconductor Technology. RCA Rev. 31, 187–206.

[B21] Kunze A. Bally M. Höök F. Larson G. (2013). Equilibrium-fluctuation-analysis of Single Liposome Binding Events Reveals How Cholesterol and Ca^2+^ Modulate Glycosphingolipid Trans-interactions. Sci. Rep. 3, 1452. 10.1038/srep01452 23486243 PMC3596795

[B22] Latza V. M. Demé B. Schneck E. (2020). Membrane Adhesion via Glycolipids Occurs for Abundant Saccharide Chemistries. Biophysical J. 118, 1602–1611. 10.1016/j.bpj.2020.02.003 PMC7136278 32097623

[B23] Lay W. K. Miller M. S. Elcock A. H. (2016). Optimizing Solute-Solute Interactions in the GLYCAM06 and CHARMM36 Carbohydrate Force fields Using Osmotic Pressure Measurements. J. Chem. Theor. Comput. 12, 1401–1407. 10.1021/acs.jctc.5b01136 PMC5082696 26967542

[B24] Le Grand S. Götz A. W. Walker R. C. (2013). SPFP: Speed without Compromise-A Mixed Precision Model for GPU Accelerated Molecular Dynamics Simulations. Comput. Phys. Commun. 184, 374–380. 10.1016/j.cpc.2012.09.022

[B25] Lis L. J. McAlister M. Fuller N. Rand R. P. Parsegian V. A. (1982). Interactions between Neutral Phospholipid Bilayer Membranes. Biophysical J. 37, 657–665. 10.1016/S0006-3495(21)00385-4 PMC1328851 7074191

[B26] Lis L. J. Parsegian V. A. Rand R. P. (1981). Binding of Divalent Cations to Dipalmitoylphosphatidylcholine Bilayers and its Effect on Bilayer Interaction. Biochemistry 20, 1761–1770. 10.1021/bi00510a009 6164391

[B27] Liu F.-T. Rabinovich G. A. (2005). Galectins as Modulators of Tumour Progression. Nat. Rev. Cancer 5, 29–41. 10.1038/nrc1527 15630413

[B28] Lorenz B. Álvarez de Cienfuegos L. Oelkers M. Kriemen E. Brand C. Stephan M. (2012). Model System for Cell Adhesion Mediated by Weak Carbohydrate-Carbohydrate Interactions. J. Am. Chem. Soc. 134, 3326–3329. 10.1021/ja210304j 22296574 PMC3288207

[B29] Miyamoto S. Kollman P. A. (1992). Settle: An Analytical Version of the SHAKE and RATTLE Algorithm for Rigid Water Models. J. Comput. Chem. 13, 952–962. 10.1002/jcc.540130805

[B30] Nodet G. Poggi L. Abergel D. Gourmala C. Dong D. Zhang Y. (2007). Weak Calcium-Mediated Interactions between Lewis X-Related Trisaccharides Studied by NMR Measurements of Residual Dipolar Couplings. J. Am. Chem. Soc. 129, 9080–9085. 10.1021/ja0711056 17608422

[B31] Pincet F. Le Bouar T. Zhang Y. Esnault J. Mallet J.-M. Perez E. (2001). Ultraweak Sugar-Sugar Interactions for Transient Cell Adhesion. Biophysical J. 80, 1354–1358. 10.1016/S0006-3495(01)76108-5 PMC1301327 11222296

[B32] Poole J. Day C. J. von Itzstein M. Paton J. C. Jennings M. P. (2018). Glycointeractions in Bacterial Pathogenesis. Nat. Rev. Microbiol. 16, 440–452. 10.1038/s41579-018-0007-2 29674747

[B33] Robustelli P. Piana S. Shaw D. E. (2018). Developing a Molecular Dynamics Force Field for Both Folded and Disordered Protein States. Proc. Natl. Acad. Sci. USA 115, E4758–E4766. 10.1073/pnas.1800690115 29735687 PMC6003505

[B34] Ryckaert J.-P. Ciccotti G. Berendsen H. J. C. (1977). Numerical Integration of the Cartesian Equations of Motion of a System with Constraints: Molecular Dynamics of N-Alkanes. J. Comput. Phys. 23, 327–341. 10.1016/0021-9991(77)90098-5

[B35] Salomon-Ferrer R. Case D. A. Walker R. C. (2013a). An Overview of the Amber Biomolecular Simulation Package. Wires Comput. Mol. Sci. 3, 198–210. 10.1002/wcms.1121

[B36] Salomon-Ferrer R. Götz A. W. Poole D. Le Grand S. Walker R. C. (2013b). Routine Microsecond Molecular Dynamics Simulations with AMBER on GPUs. 2. Explicit Solvent Particle Mesh Ewald. J. Chem. Theor. Comput. 9, 3878–3888. 10.1021/ct400314y 26592383

[B37] Sauter J. Grafmüller A. (2016). Predicting the Chemical Potential and Osmotic Pressure of Polysaccharide Solutions by Molecular Simulations. J. Chem. Theor. Comput. 12, 4375–4384. 10.1021/acs.jctc.6b00295 27529356

[B38] Sauter J. Grafmüller A. (2015). Solution Properties of Hemicellulose Polysaccharides with Four Common Carbohydrate Force fields. J. Chem. Theor. Comput. 11, 1765–1774. 10.1021/ct500924f 26574386

[B39] Schnaar R. L. (2004). Glycolipid-mediated Cell-Cell Recognition in Inflammation and Nerve Regeneration. Arch. Biochem. Biophys. 426, 163–172. 10.1016/j.abb.2004.02.019 15158667

[B40] Schneck E. Demé B. Gege C. Tanaka M. (2011). Membrane Adhesion via Homophilic Saccharide-Saccharide Interactions Investigated by Neutron Scattering. Biophysical J. 100, 2151–2159. 10.1016/j.bpj.2011.03.011 PMC3149267 21539782

[B41] Schneck E. Oliveira R. G. Rehfeldt F. Demé B. Brandenburg K. Seydel U. (2009). Mechanical Properties of Interacting Lipopolysaccharide Membranes from Bacteria Mutants Studied by Specular and Off-Specular Neutron Scattering. Phys. Rev. E 80, 041929. 10.1103/PhysRevE.80.041929 19905364

[B42] Schneck E. Rehfeldt F. Oliveira R. G. Gege C. Demé B. Tanaka M. (2008). Modulation of Intermembrane Interaction and Bending Rigidity of Biomembrane Models via Carbohydrates Investigated by Specular and Off-Specular Neutron Scattering. Phys. Rev. E 78, 061924. 10.1103/PhysRevE.78.061924 19256885

[B43] Schneider M. F. Mathe G. Tanaka M. Christian Gege and C. Schmidt R. R. (2001). Thermodynamic Properties and Swelling Behavior of Glycolipid Monolayers at Interfaces. J. Phys. Chem. B 105, 5178–5185. 10.1021/jp0028103

[B44] Schneider M. F. Zantl R. Gege C. Schmidt R. R. Rappolt M. Tanaka M. (2003). Hydrophilic/hydrophobic Balance Determines Morphology of Glycolipids with Oligolactose Headgroups. Biophysical J. 84, 306–313. 10.1016/s0006-3495(03)74851-6 PMC1302612 12524284

[B45] Stoffel W. Bosio A. (1997). Myelin Glycolipids and Their Functions. Curr. Opin. Neurobiol. 7, 654–661. 10.1016/s0959-4388(97)80085-2 9384539

[B46] Tanaka M. Schiefer S. Gege C. Schmidt R. R. Fuller G. G. (2004). Influence of Subphase Conditions on Interfacial Viscoelastic Properties of Synthetic Lipids with Gentiobiose Head Groups. J. Phys. Chem. B 108, 3211–3214. 10.1021/jp0367934

[B47] Tanaka M. Schneider M. F. Brezesinski G. (2003). In-Plane Structures of Synthetic Oligolactose Lipid Monolayers-Impact of Saccharide Chain Length. Chemphyschem 4, 1316–1322. 10.1002/cphc.200300791 14714379

[B48] Tromas C. Rojo J. de la Fuente J. M. Barrientos A. G. García R. Penadés S. (2001). Adhesion Forces between Lewis X Determinant Antigens as Measured by Atomic Force Microscopy. Angew. Chem. Int. Ed. 40, 3052–3055. 10.1002/1521-3773(20010817)40:16<3052:aid-anie3052>3.0.co;2-q 12203646

[B49] Varki A. (2017). Biological Roles of Glycans. Glycobiology 27, 3–49. 10.1093/glycob/cww086 27558841 PMC5884436

[B50] Weikl T. R. Andelman D. Komura S. Lipowsky R. (2002a). Adhesion of Membranes with Competing Specific and Generic Interactions. Eur. Phys. J. E 8, 59–66. 10.1140/epje/i2002-10008-2 15010982

[B51] Weikl T. R. Groves J. T. Lipowsky R. (2002b). Pattern Formation during Adhesion of Multicomponent Membranes. Europhys. Lett. 59, 916–922. 10.1209/epl/i2002-00130-3

[B52] Witt H. Savić F. Oelkers M. Awan S. I. Werz D. B. Geil B. (2016). Size, Kinetics, and Free Energy of Clusters Formed by Ultraweak Carbohydrate-Carbohydrate Bonds. Biophysical J. 110, 1582–1592. 10.1016/j.bpj.2016.03.006 PMC4833834 27074683

[B53] Woods R. J. (2018). Predicting the Structures of Glycans, Glycoproteins, and Their Complexes. Chem. Rev. 118, 8005–8024. 10.1021/acs.chemrev.8b00032 30091597 PMC6659753

[B54] Yu Z. W. Calvert T. L. Leckband D. (1998). Molecular Forces between Membranes Displaying Neutral Glycosphingolipids: Evidence for Carbohydrate Attraction. Biochemistry 37, 1540–1550. 10.1021/bi971010o 9484224

