# Supplementary material for: Interplay of Trans- and Cis-Interactions of Glycolipids in Membrane Adhesion
Source: Front Mol Biosci. 2021 Nov 19;8:754654. doi: 10.3389/fmolb.2021.754654 (PMC8641917; doi:10.3389/fmolb.2021.754654)
Supplement: Supplementary file 1 [file Presentation1.pdf]

Supplementary Figures

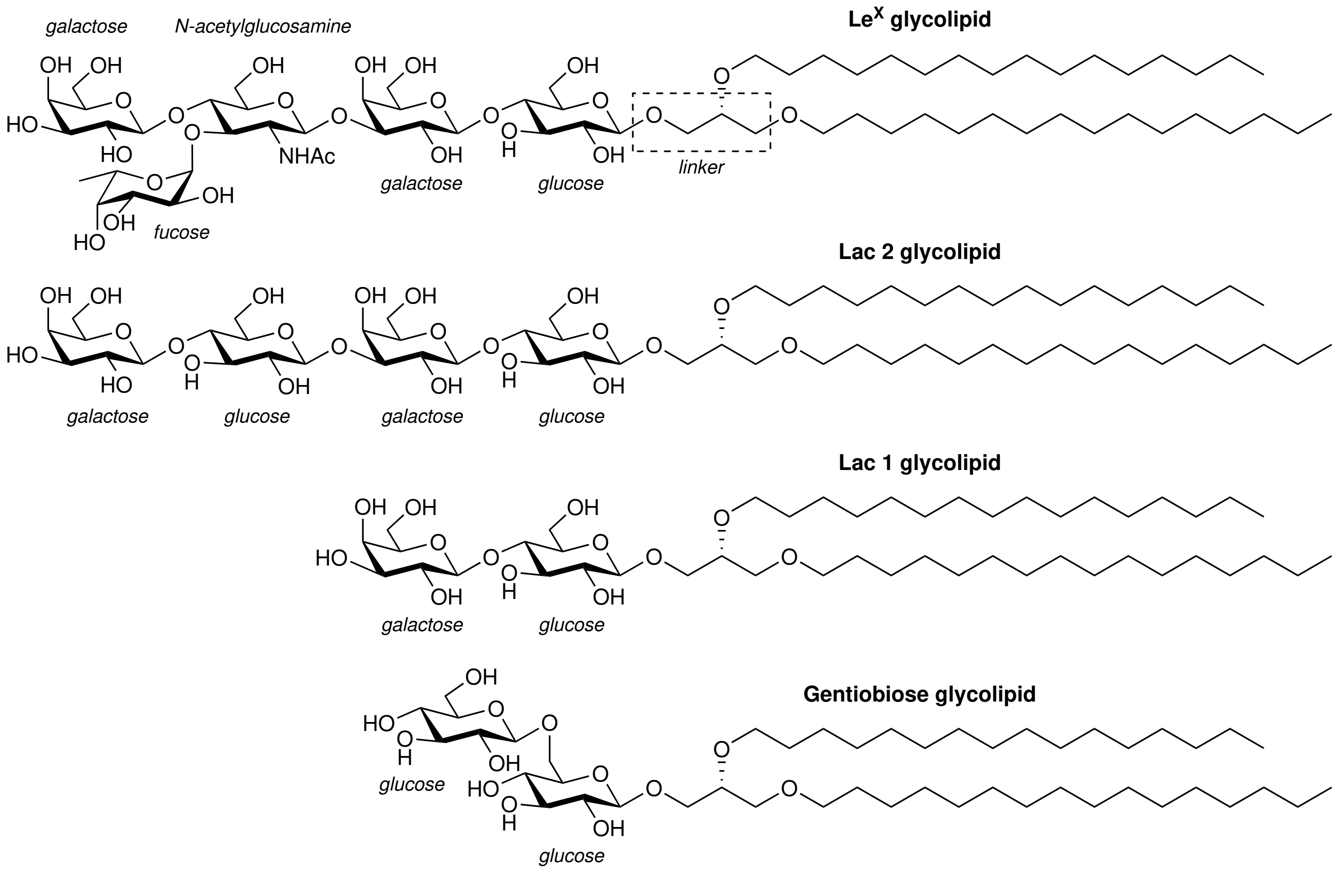

Figure S1. Structures of the glycolipids investigated in our neutron diffraction experiments.

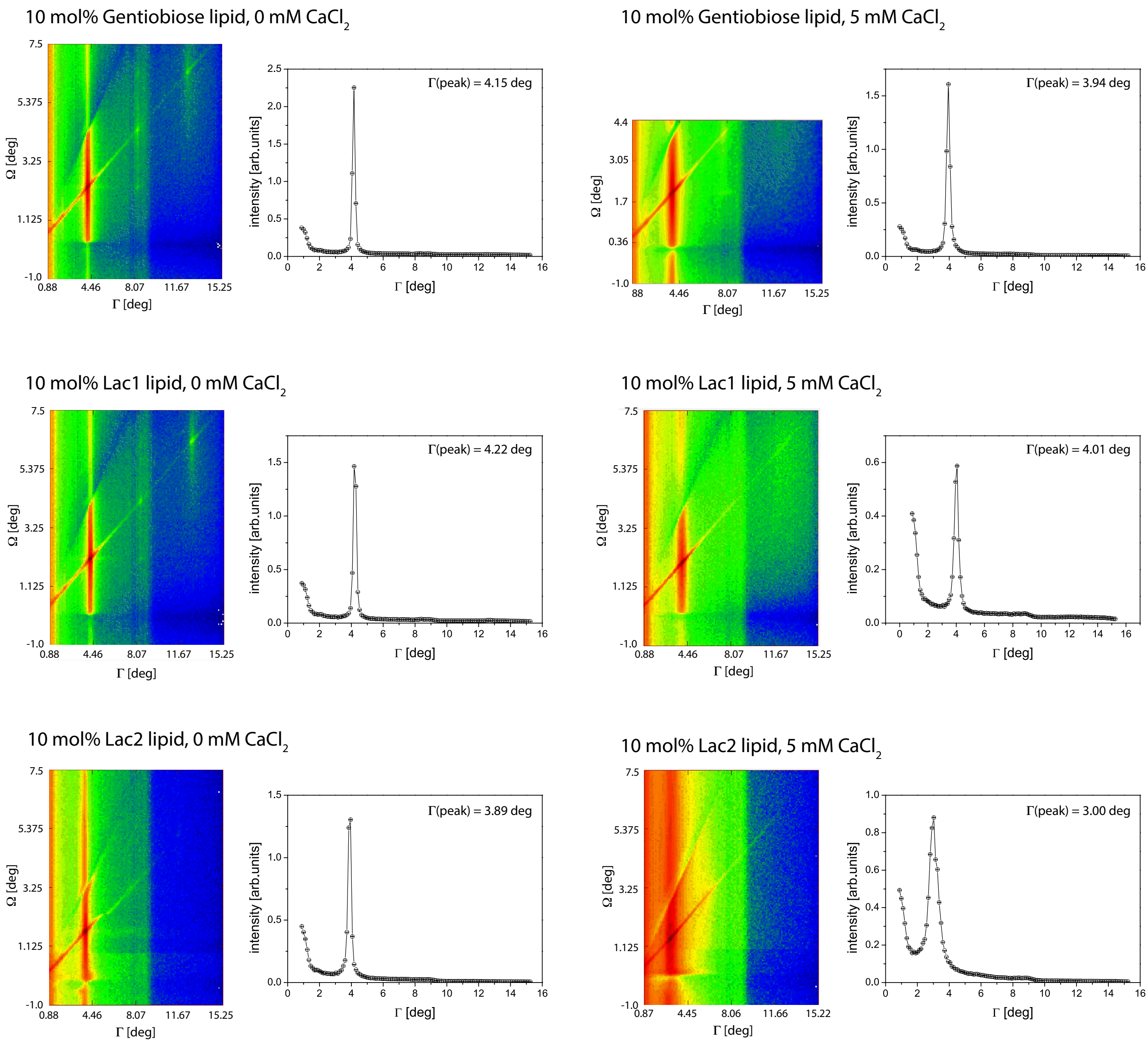

Figure S2. Overview of the neutron diffraction data on aligned multilayers of DPPC membranes doped with 10 mol% glycolipids of different types in the presence and absence of 5 mM $CaCl_2$. For each sample, the figure shows a map of the scattered intensity as a function of Γ and Ω alongside with a plot of the Ω-integrated intensity vs. Γ. The intensity maps have a logarithmic color code. Solid lines in the plots are linear interpolations to guide the eye. Peak positions were obtained through Gaussian fits to the Ω-integrated intensities in a narrow interval close to the intensity maximum.
